# Dramatic reduction of mortality in pneumococcal meningitis

**DOI:** 10.1186/s13054-016-1498-8

**Published:** 2016-10-02

**Authors:** Grete Buchholz, Uwe Koedel, Hans-Walter Pfister, Stefan Kastenbauer, Matthias Klein

**Affiliations:** 1 Department of Neurology, Klinikum Grosshadern, Ludwig Maximilians University, Marchioninistr. 15, 81377, Munich, Germany; 2 Praxis für Neurologie, Destouchesstrasse 73, 80796 Munich, Germany; 3 Emergency Department, Klinikum Grosshadern, Ludwig Maximilians University, Marchioninistr, 15, 81377 Munich, Germany

**Keywords:** Bacterial meningitis, Pneumococcal meningitis, Neurocritical care, CNS infection, Streptococcus pneumoniae, Intracranial complications

## Abstract

**Background:**

Acute bacterial meningitis is still a life threatening disease.

**Methods:**

We performed a retrospective observational study on the clinical characteristics of consecutively admitted patients with acute pneumococcal meningitis in a single tertiary care center in central Europe (from 2003 until 2015). Data were compared with a previously published historical group of 87 patients treated for pneumococcal meningitis at the same hospital (from 1984 until 2002).

**Results:**

Fifty-five consecutive patients with microbiologically proven pneumococcal meningitis were included. Most striking, mortality was down to 5.5 %, which was significantly lower than in the historical group where 24.1 % of the patients did not survive. Intracranial complications during the course of the disease were common and affected half of the patients. Unlike in the historic group, most of the intracranial complications (except ischemic stroke) were no longer associated with a low Glasgow Outcome Score at discharge.

**Conclusion:**

The drastic reduction of mortality proves there have been important advances in the treatment of pneumococcal meningitis. Nevertheless, the fact that only 44.2 % of survivors had a full recovery indicates that the search for new adjunctive treatment options must be ongoing.

## Background

*Streptococcus pneumoniae* is not only the most frequent but also one of the most threatening pathogens causing acute bacterial meningitis. Fatality rates are still high, and up to 17 % of patients die [1, 2]. Patients surviving pneumococcal meningitis often suffer from long-term neurological sequelae such as impaired neuropsychological abilities or focal neurological deficits ranging from cranial nerve palsy to hemiparesis [3–5]. Frequently, those sequelae are consequences of intracranial complications occurring during the course of the disease, such as intracranial hemorrhage, brain edema, cerebritis, empyema, sinus thrombosis, cerebral ischemia and others. Moreover, potentially fatal systemic complications such as septic shock, intravasal coagulation, acute respiratory distress syndrome and others occur frequently [6].

In a previous study from our center, 87 cases of patients suffering from pneumococcal meningitis from 1984 until 2002 were analyzed. The reported in-hospital mortality rate in that study period was 24 %. Among the patients, 75 % suffered from intracranial complications with brain edema being the most frequent (29 %), followed by hydrocephalus (16 %), arterial complications (22 %) and venous cerebrovascular complications (9 %). Only 48 % of patients had a good clinical outcome defined by a Glasgow Outcome Scale (GOS) of 5 [3]. We have since reviewed the patients’ records from the previous study to assess the current characteristics of medical care and outcome in patients with pneumococcal meningitis, beginning in 2003 and leading up to today.

## Methods

All inpatients suffering from bacterial meningitis and treated at the Neurological Department of the University of Munich from 2003 to 2015 were identified. The Neurological Department of the University of Munich is part of a tertiary care hospital (Klinikum Grosshadern) in Munich, Germany. A neurologist is present at the emergency department 24 h a day, 7 days a week. This neurologist is not involved in the patient workup as a consultant, but sees all patients with a presumed neurological diagnosis “front line”.

In the case of suspected bacterial meningitis, diagnostic workup and empiric therapy are initiated according to national guidelines (steroids were introduced after publication of the Dutch dexamethasone study [7–9]). All patients received cerebral imaging (usually cerebral computed tomography (CCT), except when there were contraindications such as suspected pregnancy), before admission to the ward to check for (i) intracranial complications and (ii) infectious foci. The neurological intensive care unit (NICU) with a capacity for 12 simultaneous patients is run by a team of neurologists and neuro-intensivists with a focus on the treatment of patients with life-threatening neurological diseases: more than 95 % of the patients primarily suffer from a neurologic disease (admission diagnosis). One main focus of the center is treatment of infectious diseases of the brain [10].

All patients with bacterial meningitis receive standard neuro-monitoring performed by a skilled nurse every hour, and this consists of evaluation of consciousness, pupil reaction, speech and limb weakness. Furthermore, a neurologist evaluates the patient neurologically four times daily. Follow-up cerebral imaging is done immediately in patients with neurologic deterioration, new-onset focal neurologic deficits or a persistent loss of consciousness without obvious reasons. Transcranial Doppler sonography is done every 48 h to screen for signs of vasculopathy in the anterior and posterior circulation [11].

In this study, only patients with acute bacterial meningitis in whom *S. pneumoniae* was detected by microbiological testing were included in the study. This included patients in whom pneumococci were identified by Gram staining and microscopy of the cerebrospinal fluid (CSF), culture from CSF, polymerase chain reaction (PCR) from CSF, CSF antigen testing or blood culture (in combination with the following CSF alterations: CSF pleocytosis, elevated CSF protein, and decreased CSF/serum glucose ratio <0.3).

Data were obtained from any medical records available. For assessment of clinical outcome at the time of discharge from our hospital, we used the GOS (with 1 = death, 2 = persistent vegetative state, 3 = severe disability, 4 = moderate disability and 5 = good recovery) [12]. A GOS of 5 was referred to as a “good clinical outcome”. A GOS of 1–4 was considered as an “unfavorable outcome”.

As the search for patients and the assessment of patient data was performed in the same way as in the previous study by Kastenbauer et al., we compared the epidemiological and clinical data from our current patient population with the data from the patients analyzed from 1984 to 2002 [3]. Figures were prepared and statistical analysis performed using Graph Pad Prism or Sigma Plot. All categorical outcomes were determined using Fisher’s exact test, whereas all continuous outcomes were compared using the Mann-Whitney test. Odds ratios (OR) with 95 % confidence intervals (in Tables 4 and 5) were calculated from 2 × 2 tables. Multivariate analysis of risk factors for death or an unfavorable outcome (GOS <5) was performed for both study periods together and for each single study period separately using multiple logistic regression. The data supporting the conclusions of this article are included within the article.

## Results

**Fig. 1:**
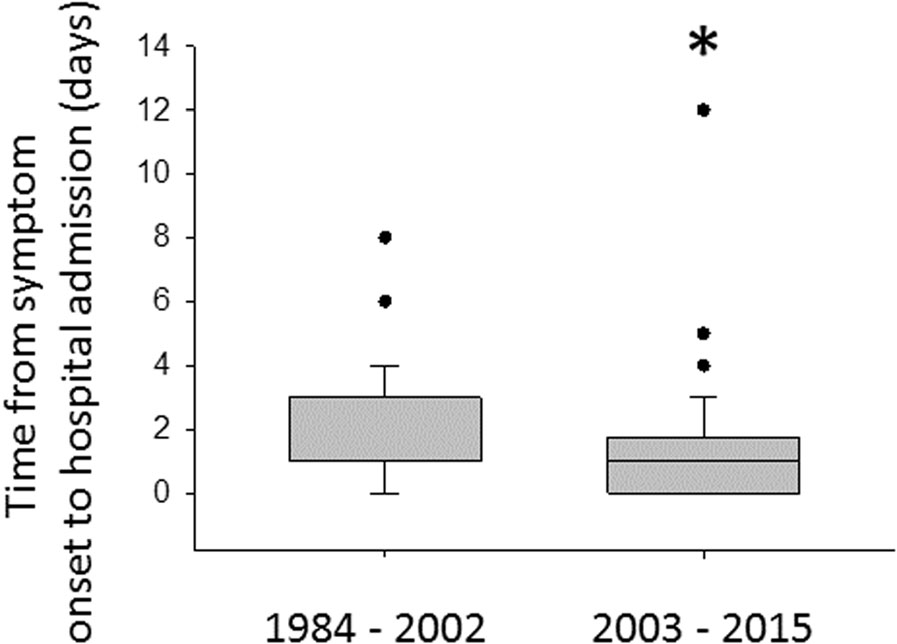
Duration of symptoms before hospital admission. Duration of symptoms in study period 1 and study period 2 until hospital admission; *p < 0.05

From 2003 to 2015, 55 patients suffering from pneumococcal meningitis were treated at our department. Of the 55 patients, 21 were admitted directly from our emergency department and 34 were transferred from another hospital. Comparing 1984 to 2002, the patients in the recent study were older and more often female (Table 1). The reported duration of symptoms before admission to the emergency department was currently lower than between 1984 and 2002 (*p* = 0.0047, see Fig. 1). Cerebrospinal fistulas were less frequently the underlying entry route of infection. All other characteristics of the currently analyzed patient group, including Glasgow Coma Score (GCS) on admission, were similar to those from 1984 to 2002. CSF laboratory findings on admission did not differ between the two groups (Table 2).

**Table 1:** Epidemiological data on admission

	2003–2015	1984–2002	*P* value
Age (median (minimum, maximum))	62 (20, 78)	51 (14, 87)	0.012
Gender (male/female)	19/36	46/41	0.039
GCS (median (minimum, maximum))	12 (3, 15)	10 (3, 15)	0.224
Fever on admission	74.1 %	96.6 %	0.0001
Neck stiffness	84.9 %	95.4 %	0.057
Chronic underlying disease	40 %	29.9 %	0.275
ENT focus	67.3 %	57.5 %	0.290
CSF fistula	5.5 %	20.7 %	0.015
Direct admission	65.5 %	59.8 %	0.5950

*GCS* Glasgow Coma Score, *ENT* ear nose and throat, *CSF* cerebrospinal fluid

**Fig. 2:**
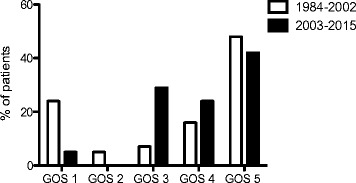
Outcome of patients with pneumococcal meningitis. Mortality (Glasgow Outcome Scale (*GOS*) = 1) was reduced in patients with pneumococcal meningitis in the current study period

**Table 2:** Laboratory findings on admission

	2003–2015	1984–2002	*P* value
Cells in CSF/μl (mean ± SD)	3411 ± 4457	3936 ± 5699	0.671
Protein in CSF in mg/dl (mean ± SD)	582 ± 851.1	458.3 ± 391.4	0.393
Glucose in CSF in mg/dl (mean ± SD)	27.33 ± 37.61	22.32 ± 23.31	0.430

*CSF* cerebrospinal fluid, *SD* standard deviation

The administered antibiotics were similar in the two study periods, as the recommended empiric therapeutic regimen given on suspicion of bacterial meningitis has not changed in Germany. In the recent study period, 52 out of the 55 patients were treated with ceftriaxone (dosage 4 g intravenously per day). Three patients were treated with meropenem and vancomycin because the medical history of these patients prompted physicians to initially expect a broader range of possible causative bacteria (two patients with CSF fistula and one patient with an eroding carcinoma of the ethmoid). There were 37 patients also treated initially with ampicillin, in case of listeria. Seven patients also received acyclovir to treat for the possibility of *Herpes simplex* virus (HSV) type 1 infection. As soon as *S. pneumoniae* was identified as the causative pathogen, the antibiotic regimen was adopted, resulting in monotherapy with ceftriaxone. Patients received additional antibiotic therapy only when additional infectious sites were identified (such as aspiration pneumonia). All pneumococcal isolates were highly susceptible to third-generation cephalosporins with a minimal inhibitory concentration (MIC) <0.5 μg/ml. The same was true for all isolates identified from patients in the previous study periods. This high rate of cephalosporine susceptibility is in accordance with nationwide surveillance data indicating more than 99 % susceptibility of pneumococci to third-generation cephalosporines [13]. The median time from arrival at the hospital to administration of antibiotics was 38 minutes (minimum 5 minutes, maximum 240 minutes) for all patients who were directly admitted to our hospital.

The percentage of patients treated with dexamethasone significantly increased to 85.5 % in 2003–2015 from 18.4 % in 1984–2002 (*p* < 0.001). This was the consequence of the results of the European dexamethasone trial that were published in 2002 [7]. For both study periods together, mortality was significantly lower in the group of patients who received steroids vs. in the group of patients who did not receive steroids (9 % vs. 30 %, *p* = 0.036). A lack of corticosteroid therapy was associated with death in all patients in both study periods (OR = 14.26 (1.58–128.72), *p* = 0.018) but not with an unfavorable outcome in multivariate analysis. There was no association between corticosteroid treatment and outcome when both study periods were evaluated separately; this can most likely be explained by the fact that the distribution of steroids was quite homogenous in both periods (rate of steroid use 85.5 % in 2003–2015 and 18.4 % in 1984–2002).

Thirty-six patients received dexamethasone at a dosage of 4 × 10 mg intravenously per day for 4 days. Four patients received a dosage of 3 × 8 mg. Three patients received hydrocortisone instead of dexamethasone for treatment of sepsis. We were only able to obtain data on the timing of dexamethasone therapy for patients who were directly admitted to our hospital. In these patients, the median time from arrival at the hospital to administration of steroids was 45 minutes (minimum 5 minutes, maximum 420 minutes). None of the patients received dexamethasone before the first administration of antibiotics.

The most common cause of pneumococcal meningitis identified in our study was sinusitis and/or mastoiditis. This was found in as many as 36 of 55 patients (65 %). Among patients who were transferred from another hospital, 80 % had ENT-focused disease. Among patients who were directly admitted to our hospital through our emergency department, 43 % had ENT-focused disease. Surgery was conducted within 24 hours of hospital admission in all patients with ENT-focused disease. Two patients with endocarditis underwent cardiac surgery.

Of 55 patients, 54 were treated at the NICU with a median stay in the NICU of 8 days (minimum 1 day, maximum 62 days). There were intracranial complications in 52.7 % of patients (Table 3). The most frequent complication was the development of brain edema (14.5 %), followed by epileptic seizures (14.5 %), cerebral ischemia (12.7 %), and hydrocephalus (10.9 %). Overall, the rate of intracranial complications was lower in the current study period than from 1984 to 2002 (*p* = 0.01). In addition to the intracranial complications assessed in the former study period, 22 patients (40 %) had vasculopathy detected by transcranial ultrasound (defined as systolic cerebral blood flow >150 cm/sec in any intracranial artery).

**Table 3:** Complications in the course of the disease

	2003–2015	1984–2002	*P* value
Intracranial complications^a^	52.7 %	74.7 %	0.010
Brain edema	14.5 %	28.7 %	0.066
Epileptic seizures	14.5 %	27.6 %	0.098
Cerebral ischemia	12.7 %	18.4 %	0.485
Hydrocepahlus	10.9 %^b^	16.1 %	0.464
Sinus thrombosis	7.3 %	9.1 %	0.766
Intracranial bleeding	3.6 %	9.1 %	0.316
Cerebral vasculopthy	40 %	not assessed	
Hearing loss	32.7 %	19.5 %	0.23
Systemic complications	34.5 %	48.3 %	0.12
Pneumonia	21.8 %	21.8 %	1.0
Sepsis	16.4 %	31.0 %	0.074
Renal failure	5.5 %	11.5 %	0.371
Disseminated intravasal coagulation	3.6 %	23 %	0.002

^a^Not including cerebral vasculopathy, as this was not assessed in the previous study period. See main text for details. ^b^Five of six patients required an external ventricular drain

There was a significant reduction in disseminated intravasal coagulopathy: every other systemic complication occurred at an unchanged frequency in both study periods. Six patients developed tetraparesis due to critical illness polyneuropathy.

Per standardized protocol at the ICU, all patients with impaired consciousness were positioned with a head up tilt of 30°, in case of an increase in intracranial pressure. Impaired consciousness was associated with hydrocephalus in six patients, five of whom received an external ventricular drain and deep sedation. Deep sedation in combination with continuous drainage of the CSF was sufficient to keep the intracranial pressure below 20 mmHg in all patients. Intubation was required in 28 patients for airway protection and/or respiratory insufficiency. Patients were normoventilated with an arterial partial pressure of carbon dioxide (paCO2) of 32–36 mmHg. The need for intubation was associated with a GOS <5 at the time of discharge from our hospital (*p* = 0.003). The systolic blood pressure was kept above 100 mmHg. There were 58 % of patients requiring vasopressors. The mortality rate and the rate of unfavorable outcomes was not statistically different in patients who required or did not require vasopressors (*p* = 0.241).

Patients were treated with nimodipine if increased cerebral blood flow was detected by transcranial ultrasound (which is routinely performed in patients with meningitis every other day during the hospital stay). Whenever nimodipine was administered, patients received an arterial line for continuous blood pressure surveillance and their arterial blood pressure was kept at 120 mmHg or higher. Antiepileptic treatment was started in patients with epileptic seizures. The following drugs were used: phenytoin (n = 4 patients), levetiracetam (n = 2 patients), valproic acid (n = 1 patient) and carbamazepine (n = 1 patient). Treatment was successful, as there were no further seizures in any of these patients.

Overall, only 3 out of 55 patients (5.5 %) died: In a 74-year-old female patient who was suffering from a progressive eroding carcinoma of the ethmoid and who did not improve after 9 days of intensive care, therapy was withdrawn in accordance with the patient’s provision and in agreement with the relatives. A 74-year-old man died only 1 day after admission with massive cerebritis. Furthermore, a 66-year-old woman died after 14 days of therapy due to multiple systemic complications (sepsis with disseminated intravasal coagulopathy, acute coronary syndrome and acute renal failure). Compared to 1984–2002, mortality was significantly lower in 2003–2015 (24.1 % vs. 5.5 %, *p* < 0.05).

Follow-up lumbar puncture (LP) was performed in 35 patients (64 %); of these, 9 patients received a repeat LP at our ICU within the first 2 days to confirm a diagnosis made at a different hospital. In those patients, CSF results had improved (decrease in pleocytosis and protein and increase in CSF/serum glucose ratio) or did not differ significantly from the first LP. In the remaining 25 patients, LP was repeated later (median 10 days, minimum 3 days, maximum 25 days), and inflammatory parameters were found to be reduced in 23 of the 25 patients.

Causative pathogens could not be isolated in any of the CSF samples obtained during repeat LP (gram stain, culture, antigen test, PCR). CSF pleocytosis had increased in only two patients: in one patient CSF pleocytosis increased from 500 cells/μl to 1909 cells/μl. As a switch was observed from granulocytic to lymphocytic pleocytosis and cultures remained sterile, the antibiotic regimen (consisting of ceftriaxone 4 g/day intravenously) was not changed. The patient improved (92 cells/μl, protein 136 cells/μl, CSF glucose 82 mg/μl on follow-up LP 10 days later). Another patient had persistent pleocytosis with persistently decreased CSF/serum glucose ratios for one month, without detection of a causative pathogen. As a consequence, he received a second ENT operation on the sphenoidal and ethmoidal sinuses, which were identified as a possible second persisting focus in addition to the initial mastoiditis for which his first operation was performed. There was improvement in the CSF parameters on subsequent follow-up LPs.

Notably, all survivors in the current patient group were discharged with a GOS of 3 or more and, thus, the reduction of mortality was not associated with an increase in patients with a GOS of 2. The rate of discharge of patients with a GOS of 5 was similar in the two study periods (Fig. 2).

A low GCS on admission and chronic underlying diseases (defined as malignancies not in remission, diabetes mellitus, abuse of alcohol, terminal renal failure, chronic hepatitis or immunosuppressive therapy) were associated with an unfavorable outcome (Table 4). On multivariate analysis, a low GCS (GCS <10) on admission was also associated with an unfavorable outcome (GOS <5) in both study periods together (OR = 3.47 (1.23–9.24), *p* = 0.13) and from 1984–2002 (OR = 3.97 (1.18–13.37), *p* = 0.026). Interestingly, this was no longer the case for the study period 2003–2015 (OR = 2.32 (0.45–11.95), *p* = 0.316).

**Table 4:** Conditions that are associated with unfavorable outcome

	GOS 5	GOS <5	OR (95 % CI)	*P* value 2003–2015
Gender (male/female)	7/16	12/20	1.37 (0.43–4.29)	0.775
Age (median ± SD)	60 ± 16.3	63 ± 12.6		0.152
Direct admission to tertiary center	56.5 %	18.8 %	0.18 (0.05–0.59)	0.005
Sinusitis	56.5 %	71.9 %	1.97 (0.63–6.07)	0.2644
Chronic underlying disease	21.7 %	53.1 %	4.08 (1.21–13.70)	0.026
Fever on admission	78.3 %	68.8 %	0.68 (0.20–2.40)	0.755
Neck stiffness on admission	91.3 %	75.0 %	0.38 (0.063–2.10)	0.442
Symptoms of meningitis before admission in days (median ± SD)	1 ± 2.65	1 ± 1.13	-	0.734
GCS on admission (median; minimum, maximum)	12; 3, 15	10; 3, 15	-	0.006
FND on admission	39.1 %	18.8 %	0.36 (0.11–1.22)	0.128
Leucocytes in serum (G/l) on admission (mean ± SD)	17.8 ± 7.74	18.67 ± 8.45	-	0.718
CRP on admission (mean ± SD)	14.73 ± 9.42	23.83 ± 11.54	-	0.007
Leucocytes in CSF/μl on admission (mean ± SD)	3623 ± 3898	3237 ± 4934	-	0.460
Protein in CSF in mg/dl on admission (mean ± SD)	432 ± 417	697 ± 107	-	0.094
Glucose in CSF in mg/dl on admission (mean ± SD)	48 ± 46	10 ± 16	-	<0.001

*SD* standard deviation, *GCS* Glasgow Coma Score, *FND* focal neurologic deficit, *CRP* C-reactive protein, *CSF* cerebrospinal fluid

**Fig. 3:**
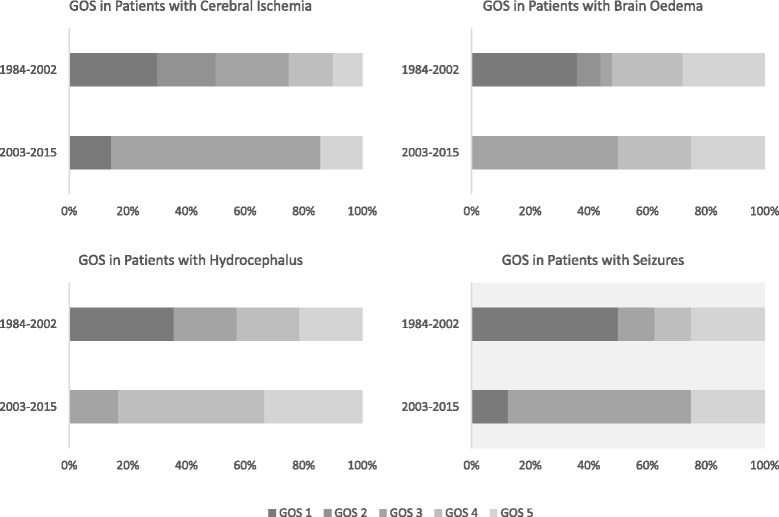
Outcome in patients with intracranial complications. For details see text. *GOS* Glasgow Outcome Scale

As in the historical patient group, the GOS in patients with intracranial complications was still worse than in those who did not develop intracranial complications (*p* = 0.011). This also held true in a multivariate analysis where intracranial complications were associated with unfavorable outcome (all patients in both study periods, OR = 11.5 (3.2–41.33, *p* < 0.001; study period 2003–2015, OR = 6.44 (1.21–34.22, *p* = 0.029)). Patients who developed a manifest ischemic stroke were discharged with a lower GOS than those who did not suffer from cerebral ischemia (*p* = 0.010). Nevertheless, the current outcome did not differ among patients who developed vasculopathy (*p* = 0.107), seizures (*p* = 0.089), brain oedema (*p* = 0.198) or hydrocephalus (0.598) in comparison to those who did not develop these complications (Table 5). This is different to the earlier study period where the development of any of these complications was associated with a low GOS (Fig. 3). Also, on multivariate analysis there was association between systemic complications and an unfavorable outcome altogether (OR = 3.57 (1.39–9.14), *p* = 0.043) and in both study periods (1984–2002, OR = 3.48 (1.04–11.63), 2003–2015, OR = 6.29 (1.21–32.79), *p* = 0.029). High serum C-reactive protein (CRP) was associated with an adverse outcome (not assessed in the former study period).

**Table 5:** Factors associated with an adverse outcome in the course of the disease

	GOS 5	GOS < 5	OR (95 % CI)	*P* value 2015
Intracranial complications				
Cerebral ischemia	4.3 %	18.8 %	5.08 (0.57–45.47)	0.219
Seizures	8.7 %	18.8 %	2.423 (0.44–13.28)	0.446
Intracranial hemorrhage	0 %	6.3 %	3.85^a^ (0.178–84.19)	0.504
Intracranial venous thrombosis	4.3 %	9.4 %	2.27 (0.22–23.41)	0.632
Brain edema	8.7 %	18.8 %	2.42 (0.44–13.28)	0.446
Hydrocephalus	8.7 %	18.8 %	2.42 (0.44–13.28)	0.446
Systemic complications				
DIC^a^	4.3 %	3.1 %	0.71 (0.04–11.98)	1.0
Sepsis	4.3 %	25.0 %	7.33 (0.85–63.49)	0.064
Pneumonia	8.7 %	31.3 %	4.77 (0.93–24.41)	0.055
Renal failure	0 %	9.4 %	5.58^b^ (0.27–113.50)	0.257

^a^These patients had thrombocytopenia, decreased antithrombin-III and clinical signs of bleeding (at sites of injections or mucous membranes). ^b^OR was calculated adding 0.5 to each value. *DIC* disseminated intravasal coagulation, *GOS* Glasgow Outcome Scale

## Discussion

The most striking finding in the study period from 2003 to 2015 is the low mortality rate of 5.5 %, representing a marked difference to the results in our earlier study from 1984 to 2002, in which 24.1 % of patients died. This result is striking: although the overall development in Europe is that case fatality rates have generally decreased in the past 20 years, such a drastic improvement has not been reported before. While 30 % of patients with pneumococcal meningitis in a prospective study in the Netherlands from 1998–2002 died, a nationwide study from the Netherlands that included more than 1400 patients admitted between 2006 and 2014 reported mortality of 18 % [1, 2]. In a study from Sweden the mortality rate was 11 % in patients suffering from pneumococcal meningitis [14]. One important reason for the improvement of outcome in this serious disease is the administration of adjunctive dexamethasone, which has been introduced in central Europe as the standard of care after the European Dexamethasone Study from the Netherlands in 2002 and the results of several meta-analyses [7, 15].

In the current analysis, intracranial complications occurred significantly less frequently than in the former study period (*p* < 0.01). The same trend was seen for systemic complications. Thus, the reduction in intracranial complications and, potentially, also in systemic complications might have contributed to the reduction in mortality. Improved outcome in patients with pneumococcal meningitis given dexamethasone has only been associated with reduced mortality due to systemic reasons, and not with reduction in intracranial complications [16]. It is impossible to draw causal pathophysiologic conclusions from clinical associations, but reduction in intracranial complications due to dexamethasone treatment is well in line with pathophysiological concepts of pneumococcal meningitis. As multiple animal studies have shown, subarachnoid inflammation triggered by bacterial compounds and danger signals is a major driver of intracranial complications, and dexamethasone therapy can lower the degree of inflammation in pneumococcal meningitis [17].

It seems difficult to attribute the very low mortality rate of 5.5 % to the introduction of adjunctive dexamethasone only. Thus, we first checked for other differences in our two populations that might have had an additional impact on mortality. One factor that might have contributed to the improvement in outcome in the recent study population could be the shorter duration of symptoms before presentation to an emergency department, as an early start of antibiotic therapy is known to contribute to a better prognosis in acute bacterial meningitis [18]. This has only been shown for the delay of antibiotics from the time point of arrival of the patient at the hospital but should also be a factor for the time period of disease duration until the patient is admitted to the emergency department.

Other differences in the study populations are less likely to be of relevance: the current patients were older, which is an unlikely factor for reduced mortality, as increased age has been reported to be an independent risk factor for death due to bacterial meningitis [19]. Second, the proportion of female patients was higher in the current study, but female gender has also been described as a risk factor for adverse outcome in the elderly with meningitis. The fact that fewer patients suffered from a CSF fistula as an underlying condition with pneumococcal meningitis is not to our knowledge known to be a risk factor for good or adverse outcome.

In addition to antibiotic therapy being started earlier and the administration of dexamethasone, treatment options in intensive care medicine have improved and might have contributed to the outcome. With knowledge on complications in pneumococcal meningitis there is an increasing chance to anticipate and detect these complications, which enables physicians to take adequate measures early. Unfortunately, most ICU interventions in pneumococcal meningitis have not been evaluated in prospective studies. Examples are interventions to decrease increased cranial pressure when suspected, including deep sedation, head tilt elevation at 30° and placement of an external ventricular drain in the case of clinically relevant hydrocephalus. In line with these data, a recent study from Sweden showed that patients with acute bacterial meningitis seem to benefit from medical care at an ICU experienced in the treatment of patients with severe neurological diseases and the possibility to undertake adequate measures (e.g., placement of an external ventricular drain) [20]. It is of great importance that hyperventilation of such patients is avoided as it lowers the intracranial pressure at the cost of reduced cerebral blood flow.

Further interventions in our patients included treatment with nimodipine and the prevention of a drop in cerebral perfusion pressure when vasculopathy has been identified by transcranial ultrasound [11, 21]. In sinus thrombosis, intravenous heparin was used (whenever the transverse sinus was not affected). Such measures have potentially dangerous side effects, especially in severely ill patients who are sedated and in whom neuromonitoring is limited. Nevertheless, the low mortality in this study and the data from Glimaker et al. [20] suggest that patients with pneumococcal meningitis benefit from treatment at centers specialized in the treatment of patients with severe neurologic diseases and we consider it likely that mortality rates at other single specialized centers in central Europe are also lower than in nationwide studies. In addition, it is a common practice at our center to perform early surgery in the case of a suspected focus of pneumococcal meningitis (e.g., sinusitis and/or mastoiditis). In recent years we have managed to surgically clear the focus of infection within 24 h in these patients. Although not evaluated in any prospective studies, we consider this as a crucial factor for outcome as it is in line with the generally accepted premise “*ubi pus, ibi evacua*”*.* However, it is completely unclear whether this has a positive effect on outcome and, to our knowledge, is not done in all centers. Therefore, clinical studies should be undertaken to address this topic.

Our study has several limitations. The data were collected retrospectively. Thus, interesting factors that might have contributed to the improved outcome could not be satisfactorily identified and, thus, were not evaluated. For example, the time to the initiation of antibiotic therapy in hospital is known to be crucial in bacterial meningitis [14, 18, 22] and it is possible that antibiotics were administered earlier in recent years than in the study period from 1984 to 2002.

In the course of the disease, especially at the intensive care unit, physicians and nurses are well-trained to optimize patient care continuously. This includes careful titration of sedation, keeping blood pressure in predefined corridors, sustain optimal mechanical ventilation, detect arising complications even before they become clinically relevant and counteract them immediately. The impact of such interventions is extremely difficult to measure, and it is impossible to assess them in a retrospective study. Furthermore, we did not assess long-term outcome: 58 % of patients were transferred to rehabilitation units and hopefully further improved. This is very likely, as a relatively large proportion of patients (25 %) were sent there with a GOS of 4 and none of the patients were discharged with a GOS of 2. Thus, the chances are high that the long-term unfavorable outcome in this study was even lower than reported.

Finally, vaccination against *S. pneumoniae* has been recommended with PCV-7 in 2006 and PCV-13 in 2009 for children in Germany. This has resulted in a decline in vaccine serotypes as the causative pneumococcal isolates from adults with pneumococcal meningitis (and an increase in serotype 23B) [23]. Unfortunately, information on serotyping of the isolates is not available in our retrospective dataset. Thus, we cannot comment on whether this change of serotype distribution might have had an impact on outcome in our patient population.

## Conclusions

In this study, the current mortality in patients with pneumococcal meningitis was 5.5 %, which is significantly lower than in an earlier study from 1984 to 2002 and the lowest reported mortality for this disease in Europe so far. In addition to the introduction of corticosteroids as routine adjunctive therapy, many other interventions performed in the ICU probably contributed to the improved outcome. As the impact of single interventions remains unclear, this study points at the necessity for a prospective evaluation of ICU interventions in pneumococcal meningitis.

## Abbreviations

CCT: cerebral computed tomography
CRP: C-reactive protein
CSF: cerebrospinal fluid
DIC: disseminated intravasal coagulation
ENT: ear nose throat
FND: focal neurologic deficit
GCS: Glasgow Coma Score
GOS: Glasgow Outcome Scale
HSV: *Herpes simplex* virus
LP: lumbar puncture
MIC: minimal inhibitory concentration
NICU: neurological intensive care unit
PCR: polymerase chain reaction

## References

[1] van de Beek D, de Gans J, Spanjaard L, Weisfelt M, Reitsma JB, Vermeulen M (2004). Clinical features and prognostic factors in adults with bacterial meningitis. N Engl J Med.

[2] Bijlsma MW, Brouwer MC, Kasanmoentalib ES, Kloek AT, Lucas MJ, Tanck MW, van der Ende A, van de Beek D. Community-acquired bacterial meningitis in adults in the Netherlands, 2006-14: a prospective cohort study. Lancet Infect Dis. 2016;16(3):339-47. 10.1016/S1473-3099(15)00430-2 26652862

[3] Kastenbauer S, Pfister HW (2003). Pneumococcal meningitis in adults: spectrum of complications and prognostic factors in a series of 87 cases. Brain.

[4] Edmond K, Clark A, Korczak VS, Sanderson C, Griffiths UK, Rudan I (2010). Global and regional risk of disabling sequelae from bacterial meningitis: a systematic review and meta-analysis. Lancet Infect Dis.

[5] Weisfelt M, van de Beek D, Spanjaard L, Reitsma JB, de Gans J (2006). Clinical features, complications, and outcome in adults with pneumococcal meningitis: a prospective case series. Lancet Neurol.

[6] Pfister HW, Feiden W, Einhaupl KM (1993). Spectrum of complications during bacterial meningitis in adults. Results of a prospective clinical study. Arch Neurol.

[7] de Gans J, van de Beek D (2002). Dexamethasone in adults with bacterial meningitis. N Engl J Med.

[8] van de Beek D, Cabellos C, Dzupova O, Esposito S, Klein M, Kloek AT, Leib SL, Mourvillier B, Ostergaard C, Pagliano P (2016). ESCMID guideline: diagnosis and treatment of acute bacterial meningitis. Clin Microbiol Infect..

[9] Pfister H, Bühler R, Eiffert H, Grabein B, Klein M, Linn J, Nau R, Salzberger B, Tumani H, Weber JR. Ambulant erworbene bakterielle Meningoeinzephalitis. AWMF Leitlinien 2016

[10] Buchholz G, Ormanns S, Pfister HW, Koedel U, Klein M (2014). Neuroinfectious diseases at a European neurological tertiary care center: one-third of patients require treatment in the neurological intensive care unit. Eur J Neurol.

[11] Klein M, Koedel U, Pfefferkorn T, Zeller G, Woehrl B, Pfister HW. Arterial cerebrovascular complications in 94 adults with acute bacterial meningitis. Crit Care. 2011;15(6):R281. 10.1186/cc10565 PMC3388646 22112693

[12] Jennett B, Teasdale G, Braakman R, Minderhoud J, Knill-Jones R (1976). Predicting outcome in individual patients after severe head injury. Lancet.

[13] Imohl M, Reinert RR, van der Linden M (2015). Antibiotic susceptibility rates of invasive pneumococci before and after the introduction of pneumococcal conjugate vaccination in Germany. Int J Med Microbiol.

[14] Glimaker M, Johansson B, Grindborg O, Bottai M, Lindquist L, Sjolin J (2015). Adult bacterial meningitis: earlier treatment and improved outcome following guideline revision promoting prompt lumbar puncture. Clin Infect Dis.

[15] Brouwer MC, McIntyre P, de Gans J, Prasad K, van de Beek D (2010). Corticosteroids for acute bacterial meningitis. Cochrane Database Syst Rev..

[16] van de Beek D, de Gans J (2004). Dexamethasone and pneumococcal meningitis. Ann Intern Med.

[17] Koedel U, Klein M, Pfister HW (2010). New understandings on the pathophysiology of bacterial meningitis. Curr Opin Infect Dis.

[18] Auburtin M, Wolff M, Charpentier J, Varon E, Le TY, Girault C, Mohammedi I, Renard B, Mourvillier B, Bruneel F (2006). Detrimental role of delayed antibiotic administration and penicillin-nonsusceptible strains in adult intensive care unit patients with pneumococcal meningitis: the PNEUMOREA prospective multicenter study. Crit Care Med.

[19] Durand ML, Calderwood SB, Weber DJ, Miller SI, Southwick FS, Caviness VS, Swartz MN (1993). Acute bacterial meningitis in adults. A review of 493 episodes. N Engl J Med.

[20] Glimaker M, Johansson B, Halldorsdottir H, Wanecek M, Elmi-Terander A, Ghatan PH, Lindquist L, Bellander BM (2014). Neuro-intensive treatment targeting intracranial hypertension improves outcome in severe bacterial meningitis: an intervention-control study. PLoS One.

[21] Southwick FS (1995). Septic thrombophlebitis of major dural venous sinuses. Curr Clin Top Infect Dis..

[22] Koster-Rasmussen R, Korshin A, Meyer CN (2008). Antibiotic treatment delay and outcome in acute bacterial meningitis. J Infect.

[23] Imohl M, Moller J, Reinert RR, Perniciaro S, van der Linden M, Aktas O (2015). Pneumococcal meningitis and vaccine effects in the era of conjugate vaccination: results of 20 years of nationwide surveillance in Germany. BMC Infect Dis..

